# Disturbance of Inorganic Phosphate Metabolism in Diabetes Mellitus: Its Relevance to the Pathogenesis of Diabetic Retinopathy

**DOI:** 10.1155/2014/135287

**Published:** 2014-03-20

**Authors:** H. Vorum, J. Ditzel

**Affiliations:** ^1^Department of Ophthalmology, Aalborg University Hospital, Hobrovej 18-22, 9000 Aalborg, Denmark; ^2^Department of Endocrinology, Center for Prevention of Struma and Metabolic Diseases, Aalborg University Hospital, Hobrovej 18-22, 9000 Aalborg, Denmark

## Abstract

Early in the progression of diabetes, a paradoxical metabolic imbalance in inorganic phosphate (P_i_) occurs that may lead to reduced high energy phosphate and tissue hypoxia. These changes take place in the cells and tissues in which the entry of glucose is not controlled by insulin, particularly in poorly regulated diabetes patients in whom long-term vascular complications are more likely. Various conditions are involved in this disturbance in P_i_. First, the homeostatic function of the kidneys is suboptimal in diabetes, because elevated blood glucose concentrations depolarize the brush border membrane for P_i_ reabsorption and lead to lack of intracellular phosphate and hyperphosphaturia. Second, during hyperglycemic-hyperinsulinemic intervals, high amounts of glucose enter muscle and fat tissues, which are insulin sensitive. Intracellular glucose is metabolized by phosphorylation, which leads to a reduction in plasma P_i_, and subsequent deleterious effects on glucose metabolism in insulin insensitive tissues. Hypophosphatemia is closely related to a decrease in adenosine triphosphate (ATP) in the aging process and in uremia. Any interruption of optimal ATP production might lead to cell injury and possible cell death, and evidence will be provided herein that such cell death does occur in diabetic retinopathy. Based on this information, the mechanism of capillary microaneurysms formation in diabetic retinopathy and the pathogenesis of diabetic retinopathy must be reevaluated.

## 1. Introduction 

Diabetic retinopathy is a leading cause of visual impairment in adults. In proliferative diabetic retinopathy, ischemia-induced pathologic growth of new blood vessels often causes catastrophic loss of vision. The pathogenesis of many of the various components of diabetic retinopathy remains poorly understood, but it is generally accepted that the development of retinopathy is multifactorial and that hyperglycemia is a very important constituent. Although large-scale population studies (Diabetes Control and Complication Trial (DCCT) [1] and United Kingdom Prospective Diabetes Study (UKPDS)) [2] have clearly shown the significance of preventative measures by patients with the guidance of their physicians, many patients still develop severe retinopathy despite considerable preventative efforts, and others who made little or no effort escape severe vascular sequelae [3]. Similarly, detailed statistical calculations of the DCCT study have revealed that the duration of diabetes (glycemic exposure) and HbA1c explained only 11% of the variation in retinopathy risk, suggesting that the remaining 89% of the risk variation is due to other factors [4]. These observations indicate that glucose or a factor closely related to glucose metabolism, such as intracellular phosphate, may be a causative factor. We have researched this concept for a number of years [5–8]. While low and high uncontrolled blood sugars give rise to easily recognizable clinical symptoms, low and high plasma inorganic phosphate remains unrecognizable or presents vague and general symptoms. Hypophosphatemia is strongly related to a decrease in intracellular adenosine triphosphate (ATP) in the aging process and in uremia. Lichtman et al. [9] conducted a detailed study of the relationship between hypophosphatemia and erythrocyte ATP in a patient with chronic renal disease and uremia who was under treatment with a low-protein diet, hemodialysis, and aluminum hydroxide gel. Erythrocyte ATP was markedly reduced during the period of hypophosphatemia. Discontinuation of aluminum hydroxide gel completely reversed the biochemical blood abnormality and restored erythrocyte ATP, and the correlation of serum inorganic phosphorous and erythrocyte ATP was close (correlation coefficient, *r* = 0.95, *P* < 0.001). It was suggested that similar reduction in ATP may occur in many other somatic cells.

## 2. The Change of Diabetes from a Metabolic Disease to a Major Cardiovascular Disorder

The advent of insulin treatment in the early 1920s dramatically changed the prognosis for diabetes patients, particularly young, insulin-dependent diabetes, but the resultant increase in longevity was confounded by an unforeseen and immense problem—cardiovascular disorders—which constitutes a significant burden on both diabetic patients and overall medical costs.

In the healthy organism, there is an optimal amount of insulin secreted in response to intake of carbohydrates, and blood sugar and plasma inorganic phosphate (P_i_) are maintained within narrow physiological ranges for both insulin-sensitive and insulin-insensitive tissues. In the diabetic organism, however, insulin treatment has modified the disease to a unique chronic condition characterized by a cellular glucose metabolism that is seldom in a steady state. In both insulin-dependent (Type 1) and noninsulin-dependent (Type 2) diabetics, abnormally high blood glucose is linked to nonphysiologic variations in plasma insulin content throughout a 24-hour period, sometimes too little, and at other times too much. During hyperglycemic-hyperinsulinemic conditions, high amounts of glucose enter into muscle and fat tissues (insulin-sensitive).

Intracellular glucose is metabolized by phosphorylation which leads to lower levels of plasma Pi and consequent negative effects on glucose metabolism in the insulin-insensitive tissues and the development of long-term cardiovascular sequelae. In addition, the kidneys are the major regulator of Pi homeostasis, a function not optimal in diabetes [6]. In the renal proximal tubular cell, glucose, Pi, and alanine (myoinositol and more), reabsorptions take place as secondary active transport processes with sodium (Na^+^) as the driving force. The favorable Na^+^ entry results from active extrusion of Na^+^ across the basolateral segment of the proximal tubular cells, energized by ATP hydrolysis and catalyzed by the Na/K-ATPase. Many findings are consistent with the concept that the inhibitory interaction among Na^+^-dependent transport processes results from shared dependence on the transmembrane electrochemical Na^+^ gradient. Accordingly, the cotransporters with sodium of one solute decrease the electrochemical Na^+^ gradient available to drive fluxes of other cotransporter solutes [10–12]. In this respect, glucose is more potent than Pi in stimulating the uptake of Na^+^ in renal microvillous vesicles [11]. Therefore, elevated blood glucose concentrations depolarize the brush border membrane for Pi and lead to lack of intracellular phosphate and hyperphosphaturia. That this relationship also is applicable to human diabetes has been shown in juvenile diabetic patients. The higher the blood glucose, and therefore the reabsorption rate of glucose, the lower the maximal phosphate reabsorption rate (T_mPO4_/GFR) [13].

## 3. Inorganic Phosphate, ATP, and Diabetic Retinopathy

Phosphorus plays a critical role in the storage, transfer, and liberation of energy in the organism as well as in the intermediate metabolism of carbohydrates, fat, and proteins. Inorganic phosphate (P_i_) is a vital component of DNA and RNA and participates both in glycolysis and oxidative phosphorylation. In glycolysis, P_i_ is a substrate for glyceraldehyde-3-phosphate dehydrogenase and stimulates the activities of hexokinase and phosphofructokinase.

Mitochondria are the power plants of our bodies, and their primary function is to manufacture adenosine triphosphate (ATP), which provides 90–95% of all cellular energy mainly by oxidative phosphorylation [14].

The concentration of plasma P_i_, thereby red cell 2,3-diphosphoglycerate and ATP levels, and oxygen delivery to tissue are closely interrelated in diabetes and other conditions (hyperalimentation and uremia) as has been clearly documented [6, 15–17].

The amount of ATP in the cell is small relative to the amount of energy that the cell needs to perform its normal work. Therefore, ATP must be constantly replenished according to the equation for oxidative metabolism, 3ADP + 3P_i_ + 1/2O_2_ + NADH → 3ATP + NAD + H_2_O, to provide a continuous supply of free energy available [18]. Therefore, it is likely that the combination of an unstable glucose metabolism and deficiencies of major substrates in ATP synthesis (P_i_ and/or oxygen) may lead to lack of intracellular ATP in noninsulin-dependent tissues (such as the endothelium and retina). The interruption of optimal ATP production for any reason might lead to cell injury and possibly cell death.

Herein, we provide evidence that such cell death occurs in human diabetic retinas and possibly in other tissues. Suggestions will be made that retinal microaneurysms likely presuppose three factors: (1) a weakness in vessel wall structures, (2) increased hydrostatic back pressure from distended venous microcirculation, and (3) deficient intracellular ATP/and or hypoxia, all of which may contribute to retinal microaneurysms and could be present in diabetes. Depletion of endothelium and pericytes may obliterate capillaries and result in a resemblance to basement membrane tubes free of these cells, leading to the so-called “acellular capillaries.” Other vascular abnormalities may be associated with these lesions, such as thickening of capillary basement membrane, blot hemorrhages, exudates, dilatation and varicosities of capillaries and venules, increased vascular endothelium growth factor (VEGF), and other growth factors that stimulate further vascular permeability and neovascularization.

## 4. Loss of Intramural Pericytes in Diabetic Retinopathy

Cogan et al. [19] in 1961 described a selective loss of intramural pericytes (IMP) from the retinal capillaries that retained their endothelium, and IMP were replaced by “ghost cells.” The authors suggested that these cells were probably involved with control of microcirculatory blood flow and therefore were of significance in the pathogenesis of diabetic retinopathy [20]. In this respect, it is of interest that IMP constrict by application of ATP [21]. Addison et al. [22] found that while IMP degeneration in the presence of intact endothelial cells is a characteristic of diabetic retinopathy, it is not specific for this condition, since similar lesions have been demonstrated in a variety of diseases such as macroglobulinemia, myelomatosis, cyanosis, and polycythemia. However, these investigators agreed that the extent of IMP loss was far less significant in nondiabetic retinopathy. Several pathological studies related to pericytes and endothelium are based on statements. However, statistical analysis was provided by Speiser et al. [23], who carefully studied the eyes of 203 patients (46 with diabetes, 46 with hypertensive arteriosclerotic vascular disease, 27 with chronic lung disease, etc.). Their results showed a specific and highly significant loss of IMP in diabetes, regardless of age, and that such a loss was associated with an increase in ghost cells, shunt vessels, and the number of microaneurysms.

## 5. Accelerated Endothelial Cell Death and Replacement in Diabetic Retinopathy

Based on electron microscopy of diabetic skeletal capillaries, Vracko first indicated that the thickened basal membrane (BM) in patients with diabetes mellitus exhibited structural characteristics that suggested the accumulation of BM was caused by repeated episodes of endothelium cell death and regeneration [24, 25]. This suggestion was based on the following findings: (1) Lamellation of BM, (2) presence of cell debris between the lamellae, (3) absence of BM thickening between pericytes and endothelial cells, and (4) presence of acellular BM cylinders in skeletal muscle interstitium.

It has been shown that the regenerating capillaries grow within the old, acellular BM tubes and then form a new second layer of BM [26]. The result is a capillary with two lamellas of BM between which cell debris may become trapped. Repetition of endothelium death and regeneration adds successive lamellas of BM and progressively narrows the capillary caliber. Thus, repeated endothelium death and regeneration with excessive thickening of BM may participate in capillary closure in diabetes. The extent of BM thickening can, like the rings in a cross-section of a tree trunk, give some idea about the number of cell generations that have occurred in a specific capillary.

Evidence of accelerated death of retinal microvascular cells in human diabetic retinopathy has been provided by Mizutani et al. [27], who compared the occurrence of cell death in diabetic and nondiabetic individuals with the terminal deoxynucleotidyl transferase-mediated dUTP nick end labeling (TUNEL) reaction, which detects preferentially apoptotic DNA fragmentation. The TUNEL-positive nuclei were significantly greater in the microvessels of diabetic (13 ± 12 per one sixth of retina) versus control subjects (1.3 ± 1.4: *P* < 0.0016), as were the counts of TUNEL-positive pericytes and endothelial cells (*P* < 0.006). Neural retinas from both diabetic and nondiabetic subjects were uniformly TUNEL negative.

Also of interest is the study of Martin et al. [28], who found a reduced number of cell generations in fibroblasts in culture from a juvenile diabetic patient, a finding later confirmed in a larger study [29].

Measurements of total basal lamina width in skeletal muscle capillaries are another useful parameter to compare. Many studies have clearly shown that BMs, at least in some anatomical areas in some diabetic patients, are much thicker than in nondiabetics of comparable ages. The findings also showed that thickened and normal BMs are not structurally homogeneous. According to the studies of Vracko [30], this may imply that the rate of cell renewal is accelerated in diabetes, that diabetics are exposed to appropriate cell injuries at a frequency and/or intensity greater than nondiabetic controls, or that the cells of diabetics are exceptionally vulnerable to injury. However, that these findings are related to a factor in the diabetic milieu rather than a hereditary factor is supported by studies of capillary BM in identical twin pairs discordant for Type 1 diabetes in which the BM of the quadriceps muscle capillary was thicker in the diabetic than in the nondiabetic twin [31].

## 6. Mechanism of Capillary Microaneurysm Formation in Diabetic Retinopathy

Retinal capillaries consist of three structures: endothelial cells (E), basement membrane (BM), and intramural pericytes (IMP), which are located within the BM. IMP is present in the normal retina at a ratio of approximately 1 with E. Retinal microaneurysms are among the earliest visible lesions in the diabetic retina, consisting of a unilateral outpunching on the venous part of the capillaries or small venules, and particularly aggregated in the posterior polar region of the retina. Ballantyne and Loewenstein [32] made the observation that the microaneurysms are usually observed only in the two-dimensional, close-knit network of capillaries of the inner-nuclear layer. The mechanism of microaneurysm formation in diabetic retinopathy may presuppose one or more of the following factors: (1) a focal structural weakness in the vessel wall, (2) an increased hydrostatic back pressure, and (3) a focal endothelial cell proliferation. In regard to (1) a focal weakness in the vessel wall may be produced by accelerated endothelial cell death and loss of intramural pericytes. For (2) and (3) Williamson and Kilo [31], Ashton [33], and Ballantyne [34] considered that microaneurysms were caused by a chronic condition of stasis on the venous side of the retinal circulation. Such stasis would logically be produced to a greater degree in the deeper close-knit network of capillaries in the inner nuclear layer, which offer more resistance than those in the superficial plexus of the retina in venous circulatory impairment. Many ophthalmologists have been impressed by the finding of considerable venular congestion and distension prior to or simultaneous to the development of microaneurysms, particularly in young diabetics. Jütte [35] carefully measured the retinal veins and found dilations in 43 of 100 juvenile diabetics. Jütte, as well as Thiel [36], observed that the dilatation extended into the small venules and venous part of capillaries. Skovborg et al. [37] measured vessel diameters in the retina from the negatives of retinal photographs of 346 juvenile diabetics and 146 healthy subjects. The average venous diameter was 10% wider in diabetic patients than in the controls (*P* < 0.001). In diabetes, both hypophosphatemia and glycosylation of hemoglobin to HbA1c, which has increased oxygen affinity, lead to a slight left shift in the oxyhemoglobin dissociation curve that impairs oxygen release to the venous part of the microcirculation [6]. Venous dilatation (lack of tone) without associated arteriolar dilatation gives rise to a sluggish venous blood flow. With a decreasing shear rate in flow in the dilated venous microcirculation, hemorheological changes in the form of erythrocyte aggregation and blood viscosity (due to increased concentration of plasma fibrinogen and alpha_2_ globulin) are aggravated [38] and further decrease venous blood flow and increase hydrostatic back pressure, leading to stagnant hypoxia. This static condition can extend into the two-dimensional, close-knit network of capillaries of the inner nuclear layer. Increased endothelium proliferation, probably caused by IMP death, may also occur [39, 40]. Capillary remodeling during stasis, increased hydrostatic pressure, hypoxia, and local endothelium proliferation could be the mechanism for retinal microaneurysm formation. Microaneurysms are only one variety of ecstasies that occur in the retinal capillaries. Sometimes relatively long segments are so dilated they form sausage-shaped loops, as shown by Ballantyne and Loewenstein [32] who described microaneurysms derived from such varicose loops.

## 7. Vascular Aging in Heath and Diabetes

Thickening of BM also occurs with age in healthy persons but is far more common and severe in diabetes patients of similar ages. However, comparison of the macro- and microvascular lesions in diabetes with those of normal aging show morphologic similarities to such a degree that common pathogenetic mechanisms must be considered [8].

Atherosclerosis in the diabetic population tends to occur at an earlier age and with greater severity than in the nondiabetic population. Until now, there has been no convincing qualitative difference between atherosclerosis in diabetics and nondiabetics [41]. “Blinded” morpho-metric/biomicroscopic studies of small blood vessels (arterioles, capillaries, and venules) in an asymptomatic surface area (bulbar conjunctiva) of diabetic and healthy persons of various ages also showed degenerative changes at an earlier age and to more significant degrees in diabetic patients compared with controls [42]. Other than age, diabetic patients showed changes more characteristic of, but not specific to, the disease, including tortuous elongation of venous capillaries, elongation and distension of the venules extending into the venous capillaries, markedly decreased linear rate of venular blood flow, and evidence of increased plasma permeability. Rabini et al. [43] studied ATP content in human erythrocytes during aging in healthy and diabetic subjects and found a significant negative correlation between age and red cell ATP in healthy control subjects (*r* = 0.82; *P* < 0.001). They concluded that their data indicated an aging-related reduction in the erythrocyte ATP content in both healthy and probable in Type 2 diabetic subjects. Short et al. [44] studied mitochondrial ATP production in skeletal muscles during aging in 146 healthy men and women aged 18–89 yrs and demonstrated that muscle ATP production declined with advancing age. It has been shown that the concentration of plasma  P_i_  and renal tubular reabsorption of P_i_ (T_mPO4_/GFR) are similarly closely related to age and gender, with the highest values occurring in childhood. In adults, plasma P_i_ in men declines with age almost linearly to the eighties, whereas, in women under the age of 45, the values overlap those of men and then increase between 45 and 54 years before declining thereafter [45].

Thus, during aging, hypophosphatemia may also be associated with a decreasing amount of intracellular tissue ATP.

## 8. Comments

The present paper stresses the importance of lack of high energy phosphate (ATP) and hypoxia in the pathogenesis of diabetic retinopathy. This hypothesis may be strengthened by the finding that lifestyle diseases and cardiovascular risk factors are similarly associated with deficiencies of major substrates in ATP synthesis, that is, hypophosphatemia and/or lack of oxygen. Age, male gender, hypertension, obesity, metabolic syndrome, and diabetes mellitus are all associated with hypophosphatemia. In addition, smoking, hyperchylomicronemia, hypertension, and diabetes may involve defects in tissue oxygen delivery [8]. Two potential risk factors, infectious diseases and stress, may be added. Håglin et al. [46] reviewed 967 patients (449 women and 518 men) treated in 1993 at the Department of Infectious Diseases, Umeå University, Sweden. Of those, 402 (42%) showed hypophosphatemia at admission and a multiple logistic regression showed a 4-fold higher risk of low serum phosphate in patients with severe infections. Although stress is difficult to define, it is generally considered as a physical, mental, or emotional strain or tension with fight-or-flight response resulting in the release of catecholamines, especially epinephrine (adrenaline). Epinephrine is a known hypophosphatemic hormone in man [47]. The remarkably close correlation found by Lichtman and coworkers between hypophosphatemia and erythrocyte ATP [9] and its association to life style diseases and many cardiovascular risk factors makes it tempting to call hypophosphatemia “the silent killer.”

**Figure 1:**
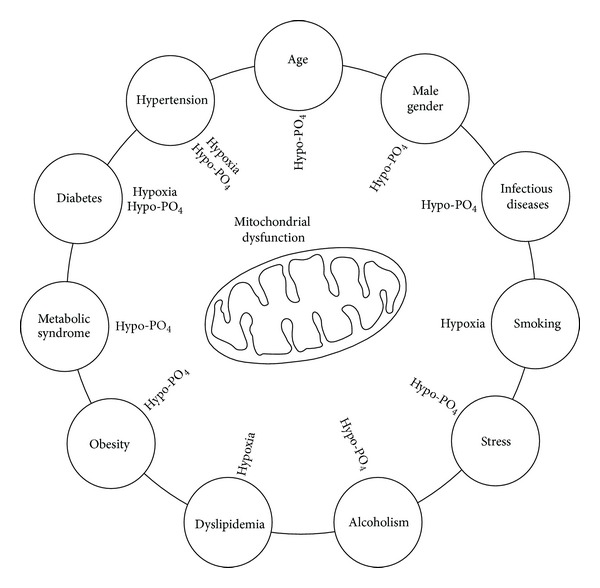
The present concept indicates that the risk factors for cardiovascular disease lead to mitochondrial dysfunction due to either hypophosphatemia and/or hypoxia (see text).

Figure 1 illustrates the concept that risk factors for cardiovascular diseases lead to mitochondrial dysfunction due to lack of intracellular phosphate and/or hypoxia.

As plasma phosphate levels decrease with age and vital tissues cannot produce sufficient ATP, the mechanism ceases to function. Like a car without gasoline, life ends when tissues cannot make sufficient ATP.

The retina has the highest oxygen demand of any tissue [48] and is therefore dependent on optimal tissue oxygen delivery and ATP production. If the endothelial cells or/and intramural pericytes lack ATP, normal vascular tone cannot be maintained and permeability will increase. As shown in Table 1, retinal capillary microaneurysms are not specific to diabetes, having been demonstrated in a number of nondiabetic clinical disorders that, however, are all associated with some type of retinal hypoxia.

**Figure 2:**
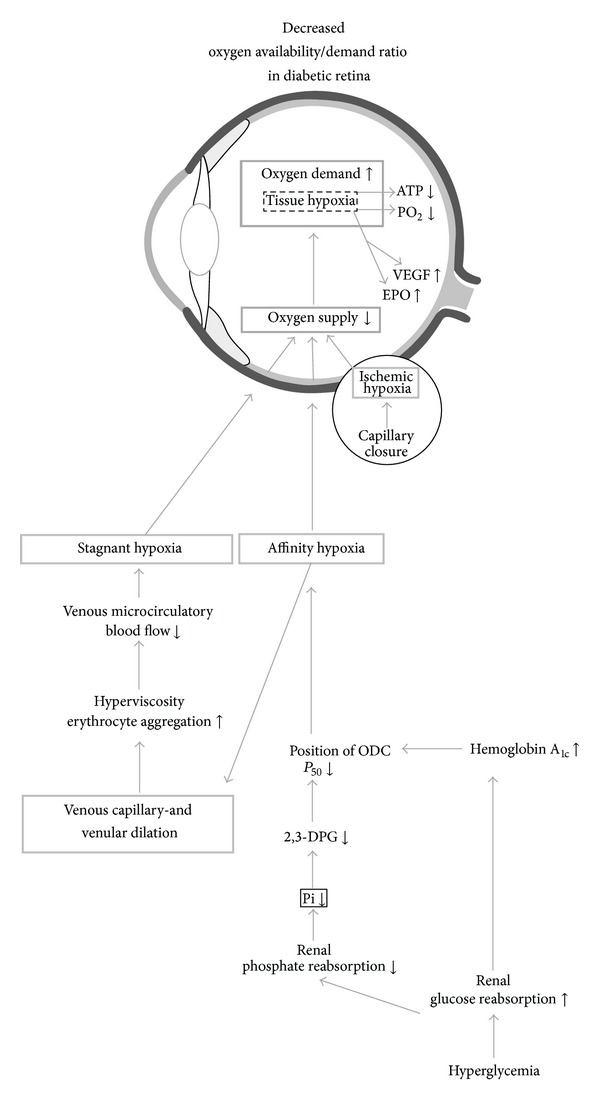
Flow chart of decreased oxygen availability/demand ratio in the diabetic retina. Hyperglycemia (lower right corner) leads to the formation of HbA_1C_ with increased binding of oxygen compared with hemoglobin A. This, combined with hypophosphatemia, leads to affinity hypoxia with decreased oxygen delivery to the venous part of the microvasculature, resulting in loss of tone in venous capillaries and venules. The resultant sluggish flow and decreased shear rate, erythrocyte aggregation, and blood viscosity (caused by increased plasma fibrinogen and alpha_2_-globulin) lead to venous microcirculatory stasis and stagnant hypoxia. Because retina has a high oxygen demand, tissue hypoxia leads to secretion of vascular endothelium growth factor (VEGF), erythropoietin (EPO), and possibly other growth factors. Capillary closure and ischemic hypoxia lead to further secretion of VEGF and other growth factors (see text).

Figure 2 shows a flow chart representing the biochemical and pathophysiological changes leading to decreased oxygen availability/demand ratio in the diabetic retina. Hypoxia is present from the early stages of diabetes as affinity and static hypoxia, possibly with secretion of vascular endothelium growth factor (VEGF) and erythropoietin (EPO). Later, when capillary closure begins, hypoxia is aggravated by ischemic hypoxia. Thereafter, further secretion of VEGF and EPO, which is also considered a potent hypoxic-inducible vascular endothelial growth factor, occurs. This latter hypoxic response to reduced oxygen availability is largely mediated by hypoxia-inducible transcription factors (HIF). High local EPO concentrations in the human vitreous body have been found to be strongly associated with proliferative diabetic retinopathy [49]. Furthermore Tong et al. [50] provided interesting data suggesting that a promoter polymorphism of the EPO gene is associated with severe diabetic eye and kidney complications. Their study suggests that a genetically determined ability of EPO synthesis predisposes diabetic patients to the development of diabetic proliferative retinopathy and end-stage renal failure.

## 9. Potential Intervention

Many questions pertaining to therapeutic interventions remain, including whether it is possible to overcome the lack of intracellular phosphate in insulin-insensitive tissues in which the vascular sequelae develop. Several therapeutic intervention trials have been carried out, including assessment of optimal glucose regulation, the effect of dietary inclusion of calcium diphosphate, and pharmacological intake of etidronate disodium, but none wholly overcome the problem [5].

## Figures and Tables

**Table 1:** Capillary retinal microaneurysms are found in the following diseases, all associated with some form of hypoxia.

Disease	Location	Mechanism
Retinal vein occlusion	Posterior, peripheral	Stagnant hypoxia
Macroglobulinemia	Peripheral, posterior	Stagnant hypoxia
Multiple myeloma	Peripheral, posterior	Stagnant hypoxia
Sickle-cell anemia	Peripheral, posterior	Stagnant hypoxia Ischemic hypoxia
Malignant hypertension	Peripheral, posterior	Ischemic hypoxia
Pulseless disease	Peripheral, posterior	Ischemic hypoxia
Diabetes mellitus	Posterior, peripheral	Affinity hypoxia Stagnant hypoxia Ischemic hypoxia
